# Quantification of energy of activation to supramolecular nanofibre formation reveals enthalpic and entropic effects and morphological consequence†
†Electronic supplementary information (ESI) available. See DOI: 10.1039/c9sc03280k


**DOI:** 10.1039/c9sc03280k

**Published:** 2019-09-16

**Authors:** Mario Samperi, Lluïsa Pérez-García, David B. Amabilino

**Affiliations:** a School of Pharmacy , University of Nottingham , University Park , NG7 2RD , UK; b The GSK Carbon Neutral Laboratories for Sustainable Chemistry , University of Nottingham , Triumph Road , NG7 2TU , UK . Email: david.amabilino@nottingham.ac.uk; c School of Chemistry , University of Nottingham , University Park , NG7 2RD , UK

## Abstract

We show a self-assembly process leading to fibres from a system that starts far from equilibrium because of fast solvent – anti-solvent mixing and analyse the activation energies associated with the aggregation. It is in some ways reminiscent of diverse natural fibrous materials that have kinetic behaviour dominated by a rate limiting induction period followed by rapid growth. A full thermodynamic rationale for these systems and related synthetic ones is required for a full understanding of the driving force of their non-equilibrium self-assembly. Here we determine quantitatively the enthalpy and entropy of activation for the processes leading to the growth of fibres of this type, that contrasts with analysis of other systems where final energetic states are analysed. A dramatic effect is revealed whereby comparatively small changes in temperature or solvent composition (the ratio of water to ethanol) lead to alterations in the relative importance of enthalpy and entropy of activation and massive changes in the speed of fibre formation. The characteristics of the kinetic model adopted show a correlation with the fibre morphology of the self-assembled materials, which are isostructural according to diffraction experiments: the control of growth can lead to fibres only two bilayers thick. The crossover in behaviour is characteristic of the solvent mixture and the thermodynamic analysis points to the origins of this effect where different assembly routes are viable under only marginally different conditions.

## Abstract 2

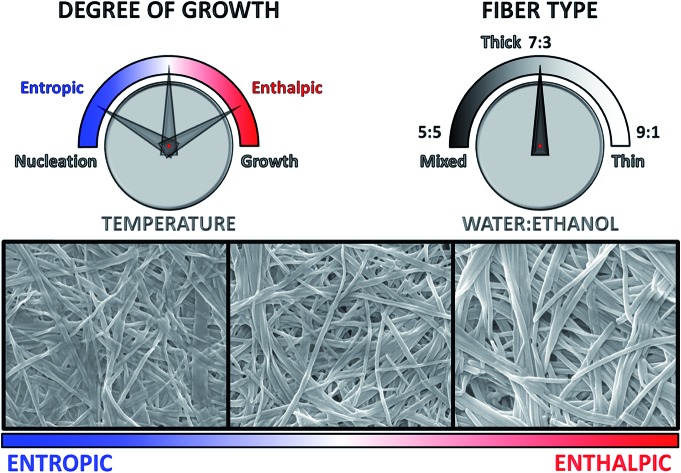
The dimensions of supramolecular fibres formed from a system that starts far from equilibrium because of fast solvent – anti-solvent mixing is determined by the balance between enthalpy and entropy in different solvent mixtures.

## Introduction

**Fig. 1:**
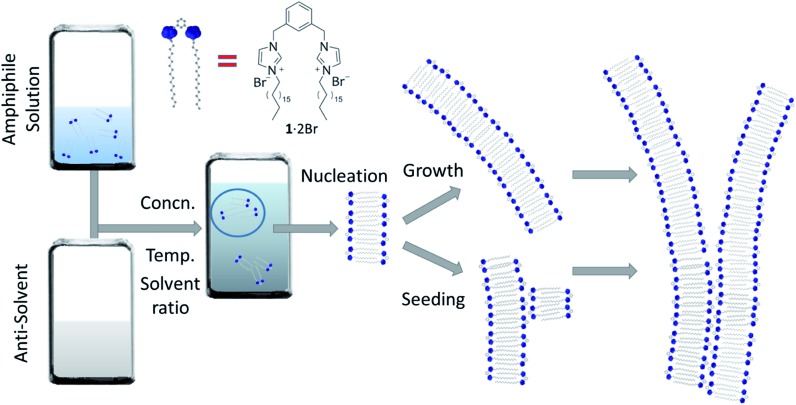
Self-assembly of nanofibres. A representation of the self-assembly process under study in this work that begins far from equilibrium: an ethanolic solution of the amphiphile (**1**·2Br) is mixed with water, at various concentrations and temperatures, and the resulting assembly nucleates at a rate dependent on the conditions. These nuclei grow leading to long fibres, and can also seed the formation of new fibres that can run parallel with the seed or branch away from it. The mathematical model we will use to fit the experimental kinetic data of fibre formation describes these phenomena, where nucleation is relatively slow, and the growth and seeding fast.

The aggregation of molecules in solution under conditions where at first the system is far from equilibrium – because of a dramatic chemical or physical changes in the medium – drives the formation of crystals, gels, nanofibres and other colloidal states,^1^,^2^ whose kinetics show certain similarities.^3^ These self-assembly processes are often dominated by the rate that they proceed at, where the system attains a particular energetic state (kinetically trapped, or metastable), whose formation can be strictly affected by experimental procedures and protocols, as a consequence of nucleation processes and multiple pathways of growth that can strongly depend on variables such as the mixing,^4^ solvent composition^5^,^6^ and temperature.^7^ Understanding the fundamental processes of the nucleation and growth in these situations can lead to controlling aggregation, such as that occurring in nature including in certain illnesses. For example, the formation of actin filaments,^8^ collagen^9^ and amyloid fibrils^10^–^12^ show self-assembly kinetics that comprise an initial induction period, where the formation of “critical seeds” or “nuclei” occurs and is the rate limiting step of the aggregation process, followed by growth. These kinds of self-assembly processes have been classified as nucleated supramolecular polymerizations,^13^ whose aggregation mechanisms exhibit cooperative characteristics and require the systems to cross a nucleation Gibbs free energy activation barrier before the aggregation becomes energetically favourable.^14^ Synthetic supramolecular systems show similar behaviour in their self-aggregation processes.^15^,^16^ Of particular relevance to the present work is the formation of self-assembled structures in aqueous solutions using water-miscible solvents, able to fully solvate the components in their monomeric form.^5^,^6^ Mixing water and solutions of the self-assembling molecules results in a big and rapid change of the chemical and physical properties of each medium, producing a quick (compared with temperature change, for example) decrease of solubility that triggers self-assembly, often under conditions far from equilibrium at the beginning of the process (Fig. 1).^17^,^18^ For amphiphiles, this process occurs by minimizing the interactions between the apolar moieties and the solvent. Solvent composition can tune the morphology of the final aggregates in these systems because of the relative importance of thermodynamic and kinetic control over the aggregation process.^19^–^21^ At relatively low proportions of water, the supramolecular organization in the aggregate is usually governed mainly by thermodynamic stability, but beyond a certain ratio of water, the process of formation of the aggregate is frozen kinetically, because of an abrupt change in molecular transport that inhibits establishment of thermodynamic equilibrium on the experimental timescale.^17^,^18^ Water-ethanol binary mixtures are one of the most commonly used among the various aqueous mixtures, because of their low toxicity, making them suitable for certain medical and biological applications from drug-delivery to tissue engineering.^22^,^23^

The non-ideal behaviour of water : ethanol mixtures is witnessed by composition variation of molar volume, surface tension, diffusion coefficient, viscosity and excess entropy.^24^ Anomalies not expected for an ideal mixture of two pure liquids are observed. This behaviour is usually attributed to a hydrophobic hydration effect, where the solvation of apolar moieties strongly influences the arrangement of water molecules close to these hydrophobic regions thus affecting the hydrogen bonding network of the solvent.^25^,^26^ Interestingly, the aggregation process of small amphiphilic molecules and block copolymers can change remarkably as function of the water : ethanol proportion, showing unusual behaviour for particular ratios and suggesting a close correlation between the self-assembly process and the properties of the solvent mixture.^17^,^27^–^30^ Quantification of these effects and understanding routes to the final structures in these complex systems are yet to be achieved.

Here, we demonstrate that changes in solvent composition and temperature during mixing determine the relative importance of enthalpy and entropy of activation of the self-assembled fibre formation in water-ethanol medium, influencing the kinetics of aggregation and directing the morphology of the material produced while maintaining the same supramolecular structure. Importantly, we determine the activation thermodynamics in a mixture that evolves rapidly to a metastable state, rather than the final thermodynamic states that are often determined in systems such as supramolecular polymers where equilibria are usually implicit.^31^–^34^ The direct link between thermodynamics of activation of fibre formation and the interlinking fibre morphology is a result of the solvent composition and temperature, and understanding this three-way relationship can guide material preparation. The implications of the observations are that the formation of materials under conditions far from equilibrium can be controlled through thermodynamic variation achieved through small changes in medium composition and temperature.

## Results & discussion

### Fibre formation and kinetic analysis

The system we explored comprises a gemini imidazolium-based amphiphile (**1**·2Br, Fig. 1) that self-assembles into supramolecular nanofibres leading ultimately to physical gels in mixtures of water and ethanol.^35^,^36^ Here we focus on the initial stages of fibre formation and the activation energies involved. The initial aggregation is triggered by addition of water to homogeneous ethanolic solutions of **1**·2Br (that are stable up to 0.2 M at 292 K); the samples are mixed quickly with a micropipette and allowed to stand undisturbed (see ESI† for details). Initially, we examined the behaviour of the system at 292 K employing various concentrations of **1**·2Br (1 to 12 mM) with five water : ethanol mixtures (5 : 5 to 9 : 1). The resulting phase diagram for different solvent ratios and amphiphile load (Fig. S1†) indicated contrasting regions for effective self-assembly, with the 7 : 3 mixture having the lowest critical gel concentration, but more importantly for us, a concentration at which all mixtures promoted significant aggregation. This preliminary analysis helped us to define three water : ethanol proportions (5 : 5, 7 : 3 and 9 : 1), where kinetic features of the assembly and ultimate fibre morphology are distinct in the different regions of the phase diagram. Thus, samples made with these three solvent ratios were explored further in this work.

**Fig. 2:**
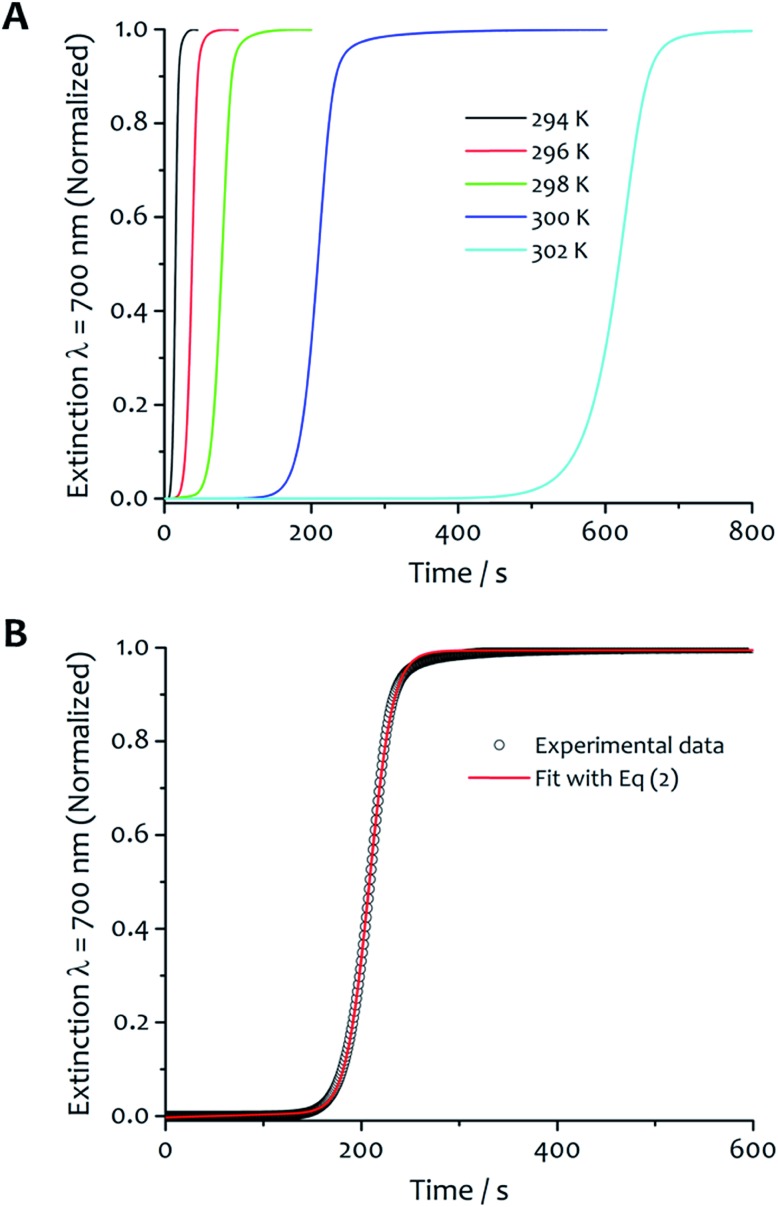
Kinetic analysis and fitting. (A) The kinetics of fibre formation at water : ethanol ratios 5 : 5 and concentration of amphiphile 8 mM at different temperatures. For this solvent composition, kinetic of fibre formation is fastest at 294 K and decreases as the temperature increases up to 302 K. (B) An example of experimental kinetic data of fibre formation in water : ethanol 5 : 5 at 300 K (black empty circles) and relative fit (red curve) with eqn (2) (*vide infra*).

The kinetics of aggregation of **1**·2Br leading to supramolecular nanofibres, that are the initial steps in the formation of a gel, were measured by using an absorption spectrometer exploiting the increased light scattering of the samples when fibrillar networks form. The kinetic profiles of fibre formation showed varying behaviours depending on temperature and solvent composition ratio, but the more common profile (observed by measuring the sample extinction at 700 nm where no absorption contribution occurs and the scattering signal is not saturated in the absorption spectrometer for all samples) comprised an induction time followed by a rapid growth and subsequent completion. This effect is seen most clearly for the case shown in Fig. 2A where the amphiphile (at a concentration of 8 mM) assembles in 5 : 5 water : ethanol displaying gradually longer induction periods and a slower aggregation process at higher temperature.

The kinetic characteristics of the assembly were obtained adopting a model developed by Pasternack proven in the analysis of aggregation of various biologically-derived and synthetic assemblies,^37^–^42^ which show a cooperative nucleated polymerization mechanism.^32^

A relative novelty in the present work is to treat the aggregation process with time dependent rate constants (rather than consecutive equilibrium steps), which reflects the increasing ‘reactivity’ of the system over time, its cooperativity and auto-catalytic behaviour. It is important to note that analyses of aggregation processes leading to linear aggregates usually employ models with a series of equilibria,^43^,^44^ which implies solving several differential equations that can only be avoided by making assumptions or approximations that finally restrict the kinetic analysis to the early portions of the kinetic profiles potentially leading to poor fits and systematic deviations between experiments and model. With the method we have used, the thermodynamic parameters (*vide infra*) derived from the evaluation of rate constants (rather than equilibria) correspond to the energies of activation to nucleation and growth. The theoretical basis of this kinetic model lies in Chaos theory and the fractal growth of self-similar clusters^45^ considering that the mean aggregate size *s*(*t*) scales as a power law dependence on time *s*(*t*) ∼ *t*^*n*^, with rate constants that are time dependent.^46^ The model implies an “active catalytic surface” (the fibre surface area) of the growing object that seeds (or “catalyses”) the formation of new “reaction centres” or “critical nuclei” containing *m* monomeric units. The formation of the critical nucleus is the rate-determining step of the aggregation process.^37^–^39^ In the present case, the growth of fibres, the process can be considered a seeding whereby the first formed fibres aid the nucleation and growth of others, with kinetic characteristics of an auto-catalytic system showing heterogeneous nucleation or secondary nucleation on the fibre's surface.^31^ The rate constant comprises both catalysed (*k*_c_) and non-catalysed (*k*_0_) pathways and is defined as follows:

1 *k*(*t*) = *k*_0_ + *k*_c_(*k*_c_*t*)^*n*^

The factor *n* is time-dependent, and describes the growth rate of the activating surface. The complete form of the integrated rate law for this model as applied to our data is the following:

2 Ext_*t*_ = Ext_∞_ + (Ext_0_ – Ext_∞_)/(1 + (*m* – 1) × {*k*_0_*t* + (*n* + 1)^–1^(*k*_c_*t*)^*n*+1^})^1/(*m*–1)^

where Ext is the extinction of the solution. For the limiting case in which the seed size (*m*) is one, which indicates the “solute species undergoing a transformation rather than the formation of a multi-molecular seed”,^47^ the above equation becomes a simpler “stretched exponential” (in this abridged form somewhat similar to the Avrami equation^48^ for phase changes that has been used for the study of isothermal gelation^49^–^51^):

3 Ext_*t*_ = Ext_∞_ + {(Ext_0_ – Ext_∞_)[1 – exp(–*kt*)^*n*^]}

This latter case corresponds to a diffusion-limited aggregating system where bigger clusters are primarily growing by reaction with smaller aggregates, producing a monodisperse system and requiring *n* < 1.^40^,^41^,^45^

The experimental kinetic data of fibre formation were fitted using eqn (2) and analysed as function of both solvent composition and temperature; an example of the fitting is shown in Fig. 2B. This analysis provided two rate constants: one corresponds to the non-cooperative formation of seeds (*k*_0_), that has relatively small values; the second (two orders of magnitude greater than *k*_0_) corresponds to the faster pathway with cooperative characteristics (*k*_c_). The parameters obtained from the fit are reported in Table S1.† The shallow slopes of the initial part of the kinetics (induction period) are reflected in the low values obtained for *k*_0_ (from 5.1 × 10^–4^ to 8.1 × 10^–6^ s^–1^), which corresponds to the non-catalysed nucleation step. The *k*_c_, rate constant for the catalytic pathway, shows values two order of magnitude higher than *k*_0_ (from 8.6 × 10^–2^ to 1.9 × 10^–3^ s^–1^), and represents the dominating pathway for this solvent mixture for all the temperature range analysed. The values of both constants follow the same trend in general, so that they decrease as the temperature increases, which is presumably a result of the lower degree of supersaturation of the amphiphile under these conditions.

**Fig. 3:**
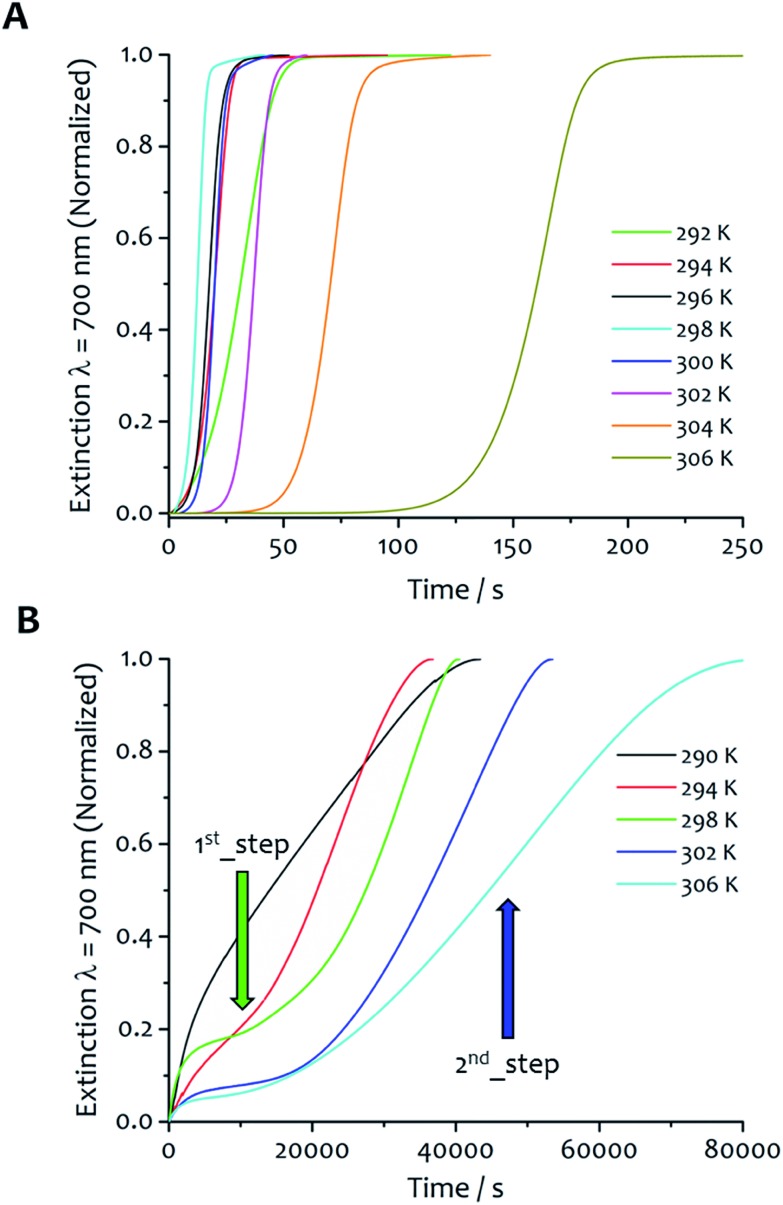
Effect of solvent ratio and temperature on kinetics. The kinetics of fibre formation at water : ethanol ratio 7 : 3 (A) shows slower aggregation at 306 K, fastest at 298 K and a less rapid self-assembly upon further decrease of the temperature from this maximum rate. (B) Kinetics of fibre formation for samples in water : ethanol 9 : 1 obtained at different temperatures display a different kinetic profile, showing a first aggregation step with no induction period followed by a second step with a sigmoidal-like growth.

The kinetic profiles obtained with solvent ratio 7 : 3 show similar general features to the 5 : 5 samples; an induction period that varied with temperature was followed by the growth of the aggregates (Fig. 3A). The rate constants are similar or higher in value to those obtained for solvent ratio 5 : 5 at the same temperature (except at 294 K), and highlight a faster aggregation process in line with a decrease of solubility at higher water content. The values of *k*_c_ are always higher than *k*_0_ over the temperature range analyzed (Table S2†). Although the autocatalytic pathway of growth is always predominant (*k*_c_ > *k*_0_), higher values of *k*_0_ were obtained for this solvent proportion, suggesting a more significant contribution of the non-seeded pathway to the overall aggregation process, particularly at low temperature (*vide infra*). For this water : ethanol ratio, the values of *k*_c_ display two different trends as function of the temperature. From 292 up to 298 K, the *k*_c_ gradually increases from 3.2 × 10^–2^ to 5.9 × 10^–2^ s^–1^. When the temperature is further increased, the values of *k*_c_ start to decrease down to 7.5 × 10^–3^ s^–1^ at 306 K.

For the water : ethanol ratio 9 : 1 (Fig. 3B), the kinetic profiles at different temperatures reveal several different features compared with the other solvent mixtures, and is characteristic of a hierarchical process overall. The kinetics are up to two orders of magnitude longer (20 000–80 000 seconds, see Fig. S2 in ESI† for comparison of the kinetic profiles obtained at 298 K for the three water : ethanol proportions, with the solvent mixture 7 : 3 showing the fastest fibre formation), and clearly display two different stages of growth. The first one takes place immediately upon injection; there is no induction period, rather a gradual increase of the extinction signal that initially tends to approach a first pseudo-equilibrium step. Successively, a second process with different kinetics occurs where the signal starts to gradually increase showing a sigmoidal-like behaviour. The data were analysed separating the two steps (Fig. S3†). Eqn (2) was adopted to fit the second part of the kinetic profiles, not considering the induction period. Consequently, the values of *k*_0_ and *m* from this fitting are not comparable with the others so they were not taken into account for the kinetic analysis. The first kinetic step was analysed separately with eqn (3), which does not consider an initial induction period (*k*_0_ and *m* are not present in the model) and which was successfully adopted in the literature to fit kinetic profiles of diffusion-limited aggregating systems.^40^,^41^

**Fig. 4:**
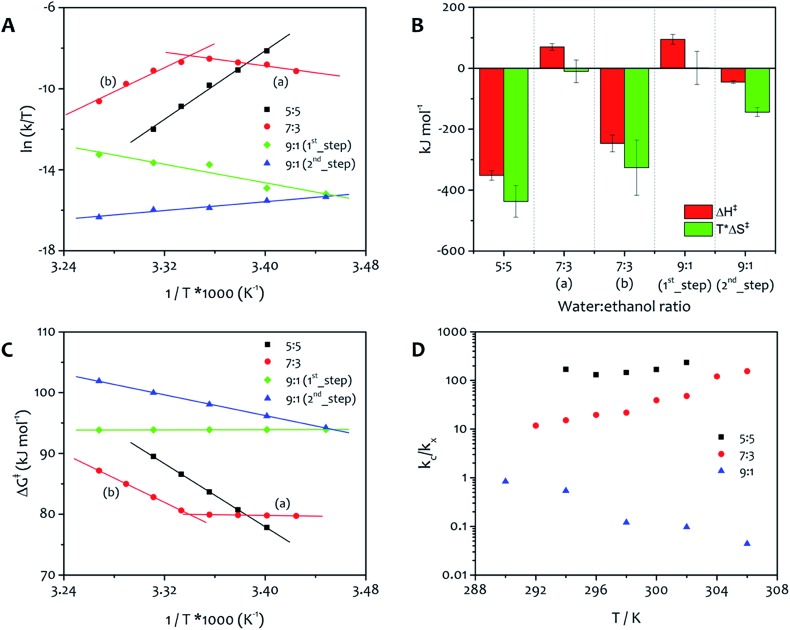
Energy of activation to fibre formation under varying conditions. (A) The fitting of the kinetic profiles of the assembly of **1**·2Br, in three different solvent ratios (water : ethanol 5 : 5, 7 : 3 and 9 : 1) and a range of temperatures, provides *k*_c_ which was fitted the Eyring equation. The Eyring plot for the 5 : 5 mixture shows a single thermodynamic regime for all temperatures studied, while the 7 : 3 mixture has two thermodynamic regimes, marked as (a) and (b). The 9 : 1 mixture has two kinetic steps (identified 1^st^ and 2^nd^) that each show a single thermodynamic regime. From the linear regions of these plots, the thermodynamic activation energy parameters can be derived, these are shown as enthalpy and entropy (reported as T*Δ*S*^‡^, with *T* = 299 K) in the bar chart (B) and the total free energy as a function of temperature (C). (D) Ratio between the catalytic (*k*_c_) and non-catalytic (*k*_*x*_) rate constant as function of temperature for the three solvent mixtures (*k*_*x*_ represents *k*_0_ for water : ethanol 5 : 5 and 7 : 3, and *k* for 9 : 1).

All the parameters obtained from the fit of these kinetic profiles are summarized in Table S3.† The first step of growth is characterized by low values of the rate constant *k* (7.3 × 10^–5^ to 5.4 × 10^–4^ s^–1^), which increase slightly with temperature. Furthermore, the values of *k*_c_ obtained from the fit of the second step are three orders of magnitude lower than those obtained for solvent ratios 7 : 3 and 5 : 5, indicating that the catalytic pathway of growth is strongly disfavoured for this solvent mixture. The derivation of *k*_c_ was made for the three representative solvent mixtures and the results were plotted in Eyring graphs that show the variation of *k*_c_ with temperature (Fig. 4A).

From the linear regions of these plots, the thermodynamic parameters for the activation energy to aggregation were calculated, allowing the relative importance of enthalpy and entropy to be determined (Fig. 4B). It is important to note that these values correspond to the activation energy to the nucleation and growth of the fibres, and the positive free energy (Fig. 4C) for all processes confirms the validity of the approach to this system, that is irreversible under the conditions used for the experiments and needs an activation barrier to be overcome before the supramolecular polymerization takes place. The absolute value of the free energy is very similar for all processes, because the product is a condensed phase of the amphiphile, in which the structure of the ordered regions is practically identical (*vide infra*). The free energy of activation corresponds to the formation of this phase, and the solvent composition apparently has a relatively small influence on this parameter. Nevertheless, the solvent ratio 9 : 1 displays the highest value of free activation energy, which reflects the relatively slow aggregation process observed for this mixture.

The Eyring plots reveal significant differences in the contribution of enthalpy and entropy to the formation of the fibres. Aggregation from 5 : 5 water : ethanol shows a single activation barrier related with *k*_c_ that has the largest enthalpic driving force (approximately 350 kJ mol^–1^) but also the largest entropic penalty of all the solvent mixtures. The reason for entropy contributing in this way for this mixture is presumably a result of the relatively low water content leading to ordered water around the polar head groups of the amphiphile. For this solvent ratio, the catalytic pathway of growth dominates the non-catalytic one. This feature can be easily seen by evaluating the ratio between the two rate constants for the process (*k*_c_/*k*_*x*_, Fig. 4D). The formation of fibres from the 7 : 3 solvent mixture displays both entropically and enthalpically determined processes, with a switchover occurring at approximately 299 K, a feature that is evident in the Eyring plot in Fig. 4A. At temperatures below 299 K (region a), enthalpy is more unfavourable and the low value of entropy facilitates the assembly, while at temperatures above 299 K (region b) the favourable enthalpic term is facilitating the assembly (a result of the favourable interactions between the alkyl chains), because entropy is a large barrier. The reason for this effect lies in the relative values of *k*_c_ and *k*_0_ obtained for this solvent mixture. The ratio between the two rate constants (Fig. 4D) is lower than the one in water : ethanol 5 : 5, and become higher as the temperature increases, suggesting that the relative importance of the two pathways (non-catalysed and catalysed) may be responsible for the switchover observed in the Eyring plot (Fig. 4A).

From the Eyring plots, it can be seen that this 9 : 1 system has separate entropically and enthalpically determined processes taking place sequentially at the same temperature, with the ratio between *k*_c_ and *k* that highlights the dominance of the first kinetic step (non-catalytic) over the second step (catalytic). The free energy of the first step, determined by enthalpy, is invariant within the temperature range whereas the second step, entropically determined, shows higher free energy values as the temperature increases (Fig. 4C). The highest free energy of activation obtained for water : ethanol ratio 9 : 1 with the lowest contribution of enthalpy is consistent with dominance of the hydrophobic effect in this mixture with the highest proportion of water.

Overall, in the Eyring graphs, the negative slopes (7 : 3 region (a) and 9 : 1 first process) correspond to assembly that is most favoured entropically. This feature indicates non-specific precipitation of the amphiphiles forming a highly solvated aggregate that does not lead immediately to gelation, a fact demonstrated most easily for the 9 : 1 water : ethanol system in which an initial colloid formation takes place (see ESI Fig. S4†). On the other hand, assembly that is determined enthalpically shows positive slopes (5 : 5, 7 : 3 (b), and second process in 9 : 1) and corresponds to a growth process where the ordered alkyl chains contribute dominantly to stabilizing the assembly.

**Fig. 5:**
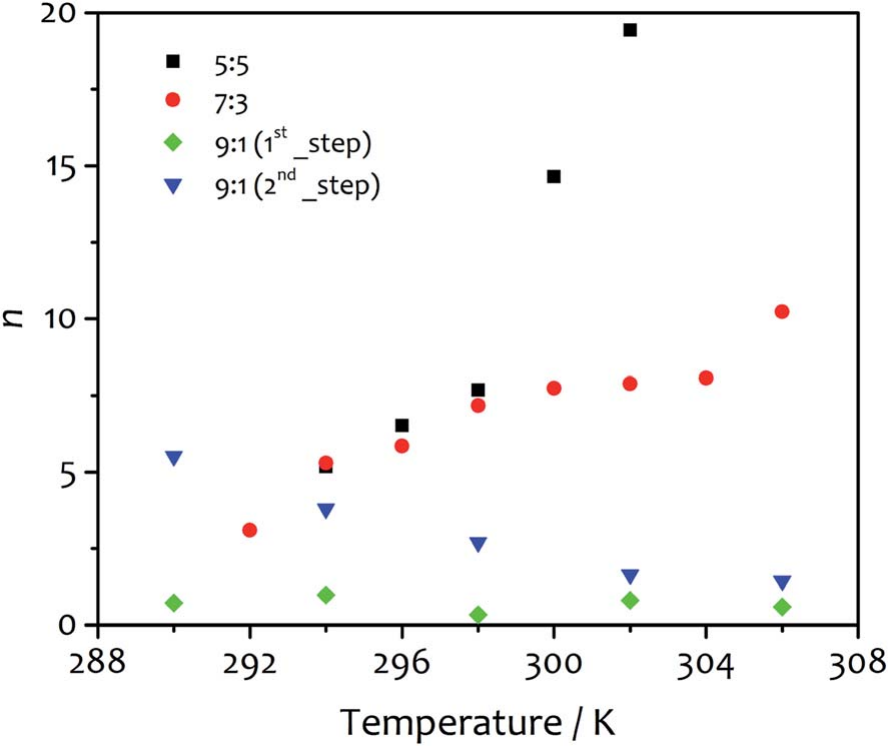
The parameter *n* obtained from the fit of the kinetic profiles as function of temperature for water : ethanol ratio 5 : 5 (black squares), 7 : 3 (red circles), 9 : 1 first step (green diamonds) and 9 : 1 second step (blue triangles). As defined by the kinetic model, this parameter describes the growth of the assembly and is related to the extension of growing surface of the aggregates, in this case the fibres.

Important information can be extracted by evaluating the parameters *m* and *n* derived from the fit of the kinetic data using eqn (2). The first one (*m*), describing the number of “monomers” participating in the rate-determining step, displays values close to unity (1.15–1.68 and 0.85–1.47 for water : ethanol ratio 5 : 5 and 7 : 3, respectively). Similar results were previously reported by Pasternack for the aggregation of insulin into amyloid fibrils.^41^ In that work, a “seed size” *m* of about one was ascribed to the protein unfolding and the conversion of a soluble monomer into an insoluble reactant unit. Similarly, our findings suggest the insolubility of the amphiphile in the new solvent mixture obtained upon water addition, which leads to de-solvation driven by hydrophilic and hydrophobic effects that promote the amphiphile aggregation. It is worth noting that our results differ from those reported for the fit of the aggregation processes of porphyrin derivatives, which suggest the formation of critical nuclei contained 3–5 monomeric units.^52^ The trends observed for *n*, the parameter related to the growth and describing the growth of the activating surface of the aggregates, find an interesting correlation with the fibre morphology obtained at different temperatures (*vide infra*). This parameter varies widely within the temperature range analysed (Fig. 5), increasing with temperature from 5.18 to 19.44 for solvent ratio 5 : 5, and from 3.10 to 10.23 for water : ethanol proportion 7 : 3. For the solvent mixture 9 : 1, the first step of growth shows values of *n* lower than one (0.32–0.97) as the model itself requires, while in the second step *n* decreases from 5.51 to 1.45 as the temperature increases. These values show some correlation with the morphology of the final gel material from the different solvent mixtures (*vide infra*).

### Fibre morphology

**Fig. 6:**
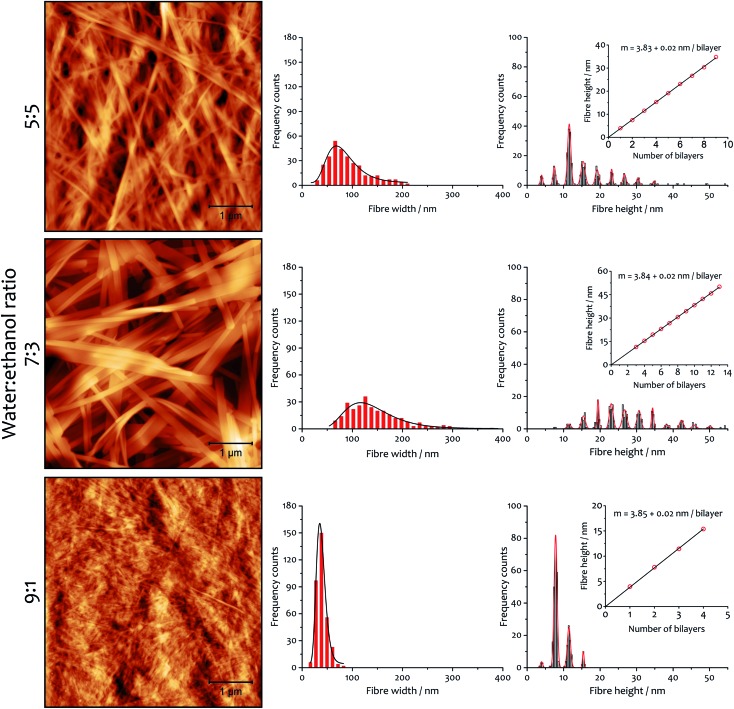
Xerogel morphology and fibre dimensions as a function of solvent composition. AFM micrographs (area 25 μm^2^) and corresponding distribution histograms of fibre width and height for xerogels made from samples 12 mM and the indicated water : ethanol ratio. In the graphs, black curves are the log-normal fit of fibre width distribution and red curves are Gaussian fits of fibre height distribution. The insets show the linear fit of Gaussian median values of the height, with slope corresponding to the thickness of a bilayer.

There are also correlations between the solvent composition, the rate of assembly and the fibrous morphology of the resulting xerogels obtained at 292 K. Atomic force microscopy (AFM) was used to quantify fibre height and width (Fig. 6), and the general morphology was observed by scanning electron microscopy (SEM, see ESI Fig. S5†). In both cases, the fibres collapse with the long axis and edge parallel to the surface on which they are deposited. The AFM analysis of fibre height clearly indicates a lamellar structure coincident with diffraction analysis (*vide infra*). A periodic distribution of height gathering around specific values (which were estimated using Gaussian profiles) dependent on solvent composition is observed.

A linear fitting of the median values (Fig. 6, insets) demonstrates the multilayer composition of the fibres, where the slope corresponds to the thickness of a single bilayer (where alkyl chains interdigitate, see below). The xerogel obtained in water : ethanol ratio 9 : 1 contains fibres comprising principally only two or three bilayers, in accord with a more transparent and weaker gel (see ESI for rheological characterization, Fig. S6, S7 and Table S5†). On the other hand, xerogels obtained with lower water content display wider distributions of thicker fibres, corresponding to 4–11 bilayers for the solvent ratio 7 : 3 and 2–6 bilayers for water : ethanol 5 : 5. The different solvent composition also affects the width of the fibres: log-normal distributions are found that are a function of the solvent proportion used. The xerogel obtained with water : ethanol ratio 5 : 5 shows 75% of the fibres having width ranging from 50 to 120 nm. When the solvent proportion was 7 : 3 fibres are noticeably wider, with 80% spanning between 80 and 210 nm. On the other hand, the xerogel obtained from the 9 : 1 mixture shows a very narrow distribution, with 90% of the fibres between 20 and 55 nm in width.

**Fig. 7:**
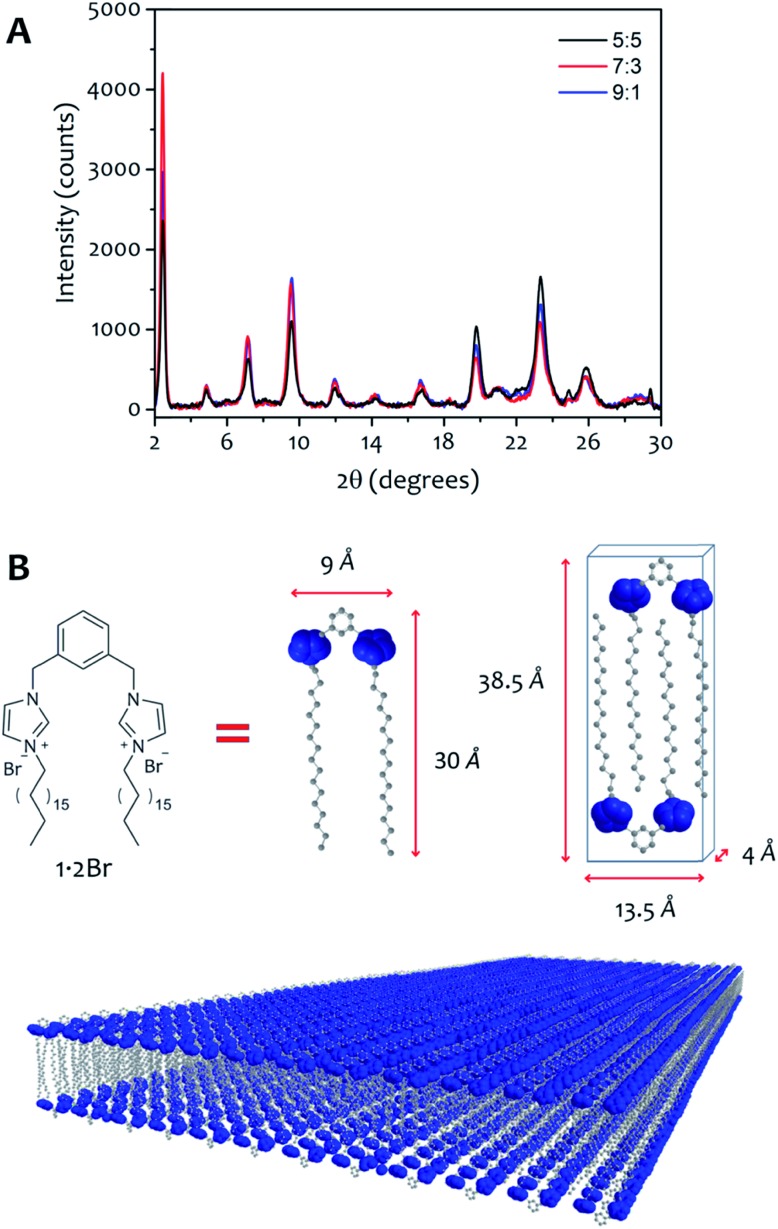
(A) Powder X-ray diffractograms of xerogel 12 mM obtained with water : ethanol ratio 9 : 1 (black line), 7 : 3 (red line) and 5 : 5 (blue line). (B) Using the approximate size of **1**·2Br, we can hypothesize a model that depicts two amphiphile molecules with interdigitated alkyl chains packed in a lamellar arrangement.

The AFM measurements suggest the same amphiphile arrangement within the fibres regardless of the water : ethanol ratio employed in the bulk liquid, which only affects the number of layers packed in a single fibre. This hypothesis was confirmed by powder X-ray diffraction experiments (Fig. 7). Xerogels made in different solvent proportions displayed similar diffraction patterns and comparable degree of crystallinity, with several peaks in the 2*θ* range between 2° and 30° that indicate a packing with two molecules of **1**·2Br with interdigitated alkyl chains in a lamellar arrangement, similarly to liquid crystals based on related compounds.^53^ This hypothesis is further supported by the data obtained from the indexing of the diffraction pattern (Table S4†). Therefore, the differing behaviour in assembly is not a result of the final supramolecular structure, but rather the self-assembly routes to the fibres.^52^

**Fig. 8:**
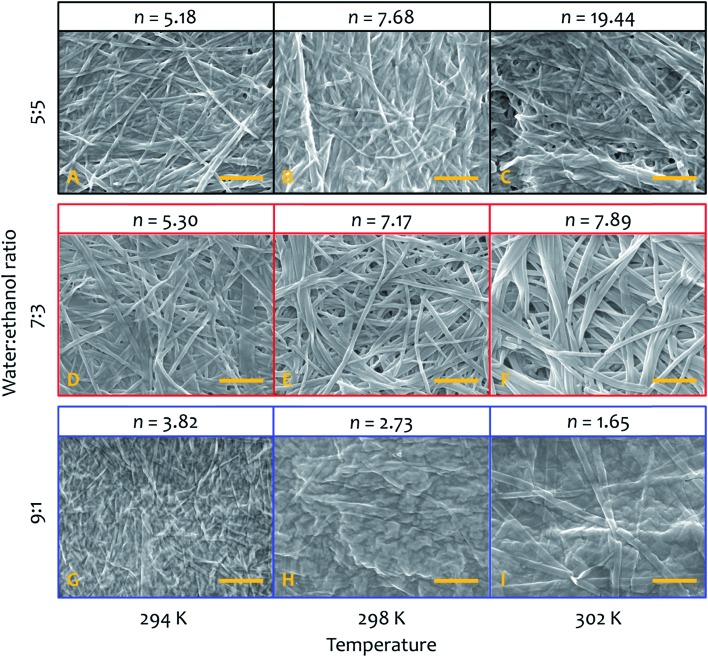
SEM micrographs (and relative value of the parameter *n* obtained from the kinetic fit of fibre formation) of xerogels made from samples at 8 mM and 294 K (left column), 298 K (central column) and 302 K (right column), in water : ethanol ratio 5 : 5 (A–C), 7 : 3 (D–F) and 9 : 1 (G–I). Scale bar in all images represents 1 μm.

SEM micrographs on xerogels made with the three solvent proportions of interest (at 8 mM of gelator, Fig. 8) at different temperatures also show significant differences in fibre morphology, and reveal a close correlation with the behaviour of the parameter *n* derived from the kinetic fitting, which describes the growth rate of the activating surface of the fibres. Generally speaking, low *n*-values (corresponding to the dominantly non-catalysed pathway) result in thin fibres, intermediate *n*-values lead to thick and wide well-defined objects. The highest *n*-values correspond to materials with clustering and bundling, in a way seen in thermally-induced gelation previously.^49^–^51^ Images acquired on xerogel obtained at 294 K in water : ethanol ratio 5 : 5 (Fig. 8A) displays a broad distribution of fibre sizes, spanning from a few to more than 10 μm in length and 50 to 150 nm in width. Fibres are quite flat and have variable morphology, which changes for the longer objects. Small groups of narrow fibres tend to run parallel to each other forming wider bundles that then diverge and join other fibres close to them, building up a highly interconnected fibrillar network with large interstitial areas, features that become much more prominent at higher temperatures (Fig. 8B and C). This finding is consistent with the higher degree of clustering observed with this solvent proportion compared to the others, and shows a close correlation with the highest values of *n* obtained from the fit (Fig. 5), the parameter related to the growth of the activating surface of the fibres. The xerogel obtained in water : ethanol 7 : 3 at 294 K (Fig. 8D) displays a different fibrillar pattern to the 5 : 5 solvent ratio, with much longer fibres between 100 and 300 nm wide. It is important to note that wider fibres clearly consist of multiple narrow fibres aligned in straight nanoribbons, tightly packed in a network with reduced interstitial regions (although clearly the collapse of the fibrous network upon drying affects the morphology, we found no evidence of lateral clustering or secondary growth upon evaporation that would be witnessed by multimodal distributions in dimensions). As the temperature increases fibres grow wider and thicker (Fig. 8E and F), but they do not cluster forming large bundles and the fibrillar network is not interconnected, observations that correlate the intermediate values of *n* obtained from the kinetic analysis for this solvent proportion (Fig. 5), suggesting a less activating surface of the fibre that does not allow growth by clustering. On the other hand, the xerogel obtained using water : ethanol ratio 9 : 1 displays quite different features (Fig. 8G). Fibres are extremely narrow (20 to 80 nm in width) with poorly defined edges and with different length and width, gathered together and intertwined in a dense fibrillar network with no interstitial areas. For this solvent ratio, temperature only partially affects the morphology of the fibres, which are moderately wider but quite thin (Fig. 8H and I, see also ESI for a SEM image of the initial growth region of these fibres, Fig. S4†). The parameter *n* obtained from the fit shows the lowest values compared to the other two solvent proportions (Fig. 5), in line with a limited activating surface of the fibres.

## Conclusions

The energy of activation to self-assembly in this non-equilibrium system is affected modestly by the ratio of water to ethanol, but the contributions of enthalpy and entropy to the processes define the kinetics and the morphology of the final material. Influencing the morphology of gel fibres by controlling the thermodynamic features of nucleation and growth could be important when considering the applicability of this kind of supramolecular gel as a drug-delivery system.^54^ Although free energy barriers are similar across the different solvent systems and temperatures, enthalpically determined processes in these systems tend to lead to the growth of larger objects (as seen in SEM images of experiments performed at different temperatures, see Fig. 8). This growth process must follow nucleation, which is a process determined mainly by entropy (and is presumably similar to supramolecular polymer formation through nucleation-elongation mechanisms^55^–^59^). These conclusions can be asserted because of the kinetic analysis and determination of the activation energies to the assembly process, all based on relatively straightforward data treatment. The kinetic model we have adopted describes well the auto-catalysis found in this cooperative supramolecular polymerization. The parameter *n* derived from the fit reflects the degree of activation exerted by the fibre's surface to the auto-catalysis or secondary nucleation, and is mirrored the morphology of the final material. Low *n*-values are observed when the non-catalysed pathway is dominant, giving rise to thin fibres. Intermediate *n*-values take account for a higher degree of activation of the fibre's surface (higher contribution of the auto-catalytic pathway to the overall process), which results in thick and wide well-defined objects, which finally tend to cluster and bundle when the degree of activation shows the highest values and the dominance of the auto-catalysis. Also, the controlled growth can lead to the formation of extremely thin fibres, only two bilayers thick. The strategy employed here could well enlighten a whole range of physico-chemical phenomena involving the aggregation of molecular and macromolecular systems for supramolecular materials^60^,^61^ that assemble under conditions far from equilibrium,^62^ quantifying energetic contributions and correlate them with assembly morphology and function.

## Conflicts of interest

There are no conflicts of interest.

## Supplementary Material

Supplementary information
