# Supplementary material for: Quantification of energy of activation to supramolecular nanofibre formation reveals enthalpic and entropic effects and morphological consequence
Source: Chem Sci. 2019 Sep 16;10(44):10256–66. doi: 10.1039/c9sc03280k (PMC6968731; doi:10.1039/c9sc03280k)
Supplement: Supplementary file 1 [file SC-010-C9SC03280K-s001.pdf]

Electronic Supplementary Material (ESI) for Chemical Science.
This journal is © The Royal Society of Chemistry 2019

Electronic Supporting Information for

Quantification of energy of activation to supramolecular nanofibre formation reveals enthalpic and entropic effects and morphological consequence

Mario Samperi,[1,2] Lluïsa Pérez-García[1] and David B. Amabilino,*[2,3]

[1] School of Pharmacy, University of Nottingham, University Park, NG7 2RD, United Kingdom

[2] The GSK Carbon Neutral Laboratories for Sustainable Chemistry, University of Nottingham, Triumph Road, NG7 2TU, United Kingdom

[3] School of Chemistry, University of Nottingham, University Park, NG7 2RD, United Kingdom

* Corresponding author: E-mail: david.amabilino@nottingham.ac.uk

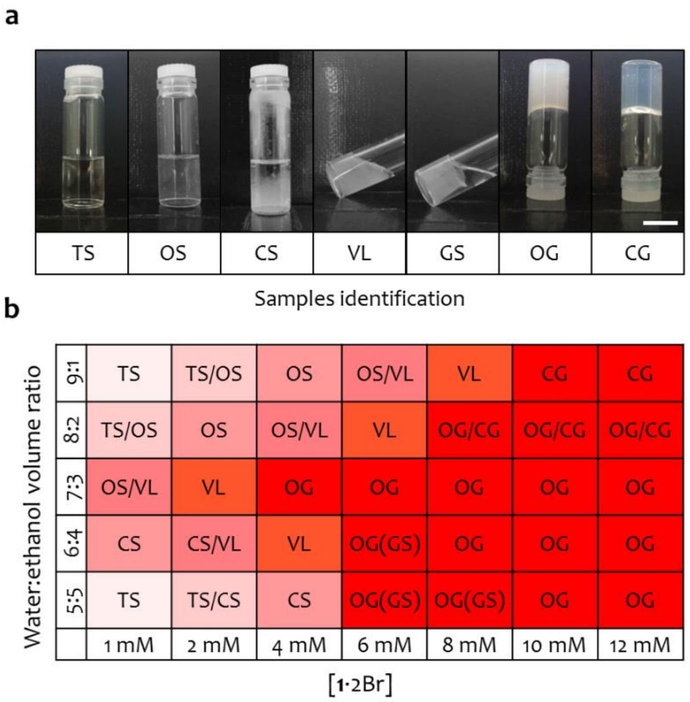

Figure S1. Phase diagram for mixtures of water, ethanol and 1·2Br. (a) Addition of water to ethanolic solutions of **1**·2Br leads to a variety of outcomes, from a transparent solution (TS), opaque solution (OS), colloidal suspension (CS), viscous liquid (VL), gel with remnant solution (exuding solvent after complete formation, GS), opaque gel (OG) and clear gel (CG). Scale bar represents 1 cm. Gelation was determined by vial inversion test. (b) The phase diagram at 292 K for varying final concentration of **1**·2Br and water:ethanol ratios indicates that the 7:3 ratio is gelled most effectively by **1**·2Br, that is, the critical gel concentration is lowest of the mixtures presented.

Results from kinetic analysis

Table S1. Values of the rate constants k_0, k_c, m and n obtained from Equation (2), for fibre formation at different temperatures in water:ethanol 5:5.

Temperature (K)	k_0 (s^{-1})	k_c (s^{-1})	m	n
294	$(5.1 \pm 1.4) \times 10^{-4}$	$(8.6 \pm 0.009) \times 10^{-2}$	1.41 ± 0.02	5.18 ± 0.03
296	$(2.6 \pm 0.2) \times 10^{-4}$	$(3.4 \pm 0.002) \times 10^{-2}$	1.29 ± 0.02	6.52 ± 0.02
298	$(1.1 \pm 0.6) \times 10^{-4}$	$(1.6 \pm 0.0004) \times 10^{-2}$	1.23 ± 0.03	7.68 ± 0.01
300	$(3.4 \pm 0.2) \times 10^{-5}$	$(5.7 \pm 0.001) \times 10^{-3}$	1.68 ± 0.01	14.65 ± 0.05
302	$(8.1 \pm 0.2) \times 10^{-6}$	$(1.9 \pm 0.0001) \times 10^{-3}$	1.15 ± 0.003	19.44 ± 0.03

Table S2. Values of the rate constants k_0, k_c, m and n from Equation (2), for fibre formation at different temperatures in water:ethanol 7:3.

Temperature (K)	k_0 (s^{-1})	k_c (s^{-1})	m	n
292	$(2.7 \pm 0.00005) \times 10^{-3}$	$(3.2 \pm 0.00005) \times 10^{-2}$	0.99 ± 0.001	3.10 ± 0.00006
294	$(2.9 \pm 0.08) \times 10^{-3}$	$(4.4 \pm 0.001) \times 10^{-2}$	1.07 ± 0.004	5.30 ± 0.01
296	$(2.5 \pm 0.2) \times 10^{-3}$	$(4.9 \pm 0.02) \times 10^{-2}$	0.85 ± 0.005	5.85 ± 0.03
298	$(2.7 \pm 0.1) \times 10^{-3}$	$(5.9 \pm 0.005) \times 10^{-2}$	1.16 ± 0.02	7.17 ± 0.09
300	$(1.3 \pm 0.06) \times 10^{-3}$	$(5.1 \pm 0.003) \times 10^{-2}$	1.47 ± 0.02	7.74 ± 0.08
302	$(7.1 \pm 0.1) \times 10^{-4}$	$(3.4 \pm 0.0008) \times 10^{-2}$	1.34 ± 0.007	7.89 ± 0.03
304	$(1.4 \pm 0.08) \times 10^{-4}$	$(1.7 \pm 0.0005) \times 10^{-2}$	1.24 ± 0.007	8.07 ± 0.03
306	$(4.8 \pm 0.1) \times 10^{-5}$	$(7.5 \pm 0.00005) \times 10^{-3}$	0.99 ± 0.001	10.23 ± 0.0001

Table S3. Kinetic parameters obtained from the fitting of fibre formation kinetics at different temperatures in water:ethanol 9:1.

T (K)	1st step		2nd step			
	k (s^{-1}) [a]	n[a]	k_0 (s^{-1}) [b]	k_c (s^{-1}) [b]	m[b]	n[b]
290	(7.3 ± 0.3) × 10^{-5}	0.72 ± 0.008	(3.0 ± 0.4) × 10^{-4}	(6.3 ± 0.1) × 10^{-5}	2.75 ± 0.02	5.51 ± 0.06
294	(9.8 ± 0.2) × 10^{-5}	0.97 ± 0.003	(1.7 ± 0.01) × 10^{-5}	(5.3 ± 0.004) × 10^{-5}	1.42 ± 0.006	3.82 ± 0.02
298	(3.2 ± 0.1) × 10^{-4}	0.32 ± 0.01	(5.5 ± 0.001) × 10^{-6}	(3.8 ± 0.001) × 10^{-5}	0.34 ± 0.003	2.73 ± 0.03
302	(3.6 ± 0.01) × 10^{-4}	0.80 ± 0.005	(3.1 ± 0.001) × 10^{-6}	(3.5 ± 0.002) × 10^{-5}	0.40 ± 0.001	1.65 ± 0.002
306	(5.4 ± 0.04) × 10^{-4}	0.58 ± 0.005	(1.6 ± 0.0001) × 10^{-6}	(2.4 ± 0.001) × 10^{-5}	0.60 ± 0.001	1.45 ± 0.001

[a] From Equation (3)
[b] From Equation (2)

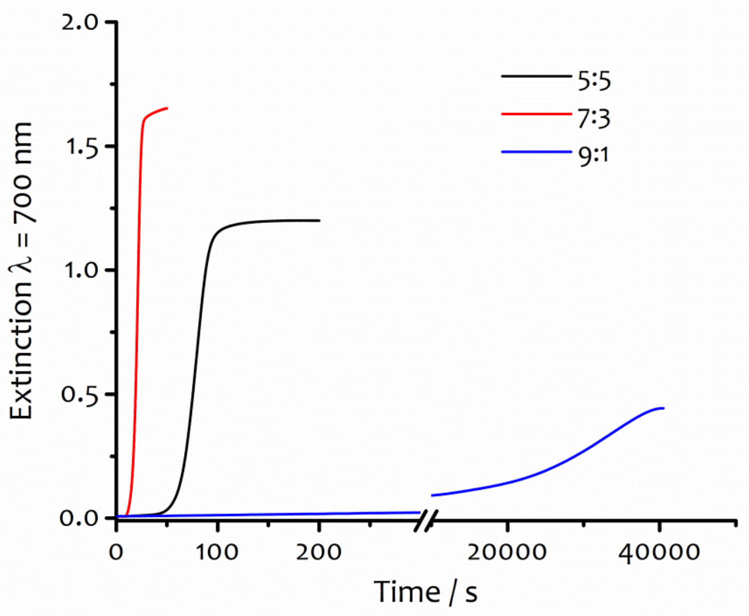

Figure S2. Comparison of kinetic profiles obtained at 298 K for water:ethanol ratio 5:5 (black curve), 7:3 (red curve) and 9:1 (blue curve), showing the 7:3 solvent mixture having the fastest fibre formation

process and the highest degree of scattering due to the thickness of the fibres formed after complete aggregation.

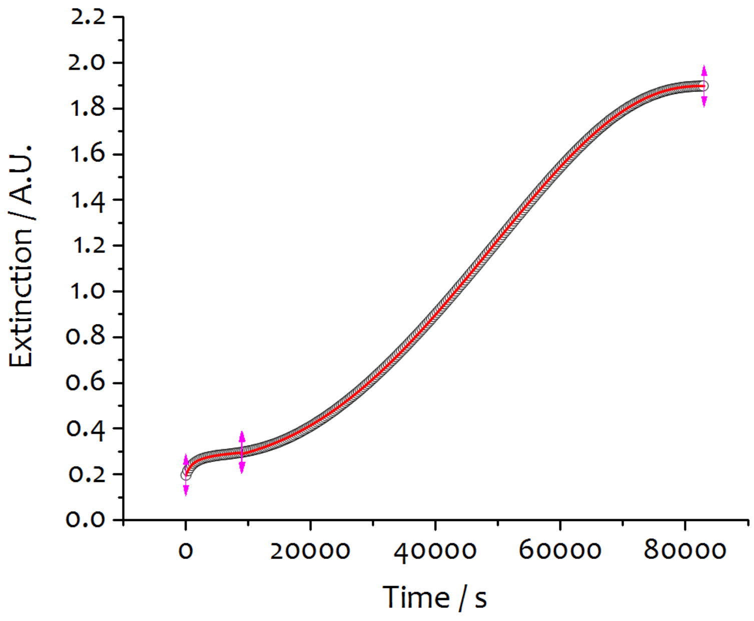

Figure S3. Example of the fit (red curves) performed on the two-steps kinetic profile (experimental data, empty black circles) obtained in water:ethanol 9:1 at 306 K. The purple arrows show the data ranges considered for the two fits, with the separation between the two steps being set at the inflection point. The first kinetic step was fitted with Equation (3) from t = 0 to the inflection point, whereas the second step was fitted with Equation (2) from the inflection point to t final. The results of the fit are reported in table S3.

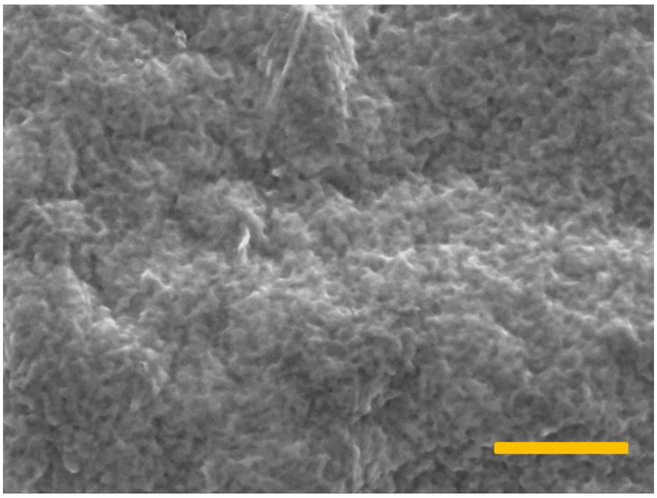

Figure S4. SEM micrographs of xerogel made from an aliquot of sample 8 mM in water-ethanol ratio 9:1 after the first kinetic step at 294 K. Scale bar represents 1 μm. A two-steps kinetic profile can suggest an initial formation of a different morphological assembly that evolves into fibres. To get further insights into the two-steps kinetic profile, the sample made at 294 K in water-ethanol ratio 9:1 was examined by taking an aliquot of solution at the end of the first kinetic step (at the inflection point where the second kinetic step starts), then a SEM analysis was performed. At this stage, the sample does not display long and defined fibres yet, rather what seems to be smaller domains of tubular-like aggregates highly intertwined. Considering that after this kinetic step, a second one occurs which leads to the formation of fibre having mainly two double layers (as shown by AFM, figure 4), this morphology could suggest that in the first kinetic step, the formation of tubular vesicles takes place which then undergo solvent extrusion from the core producing a lamellar structure.

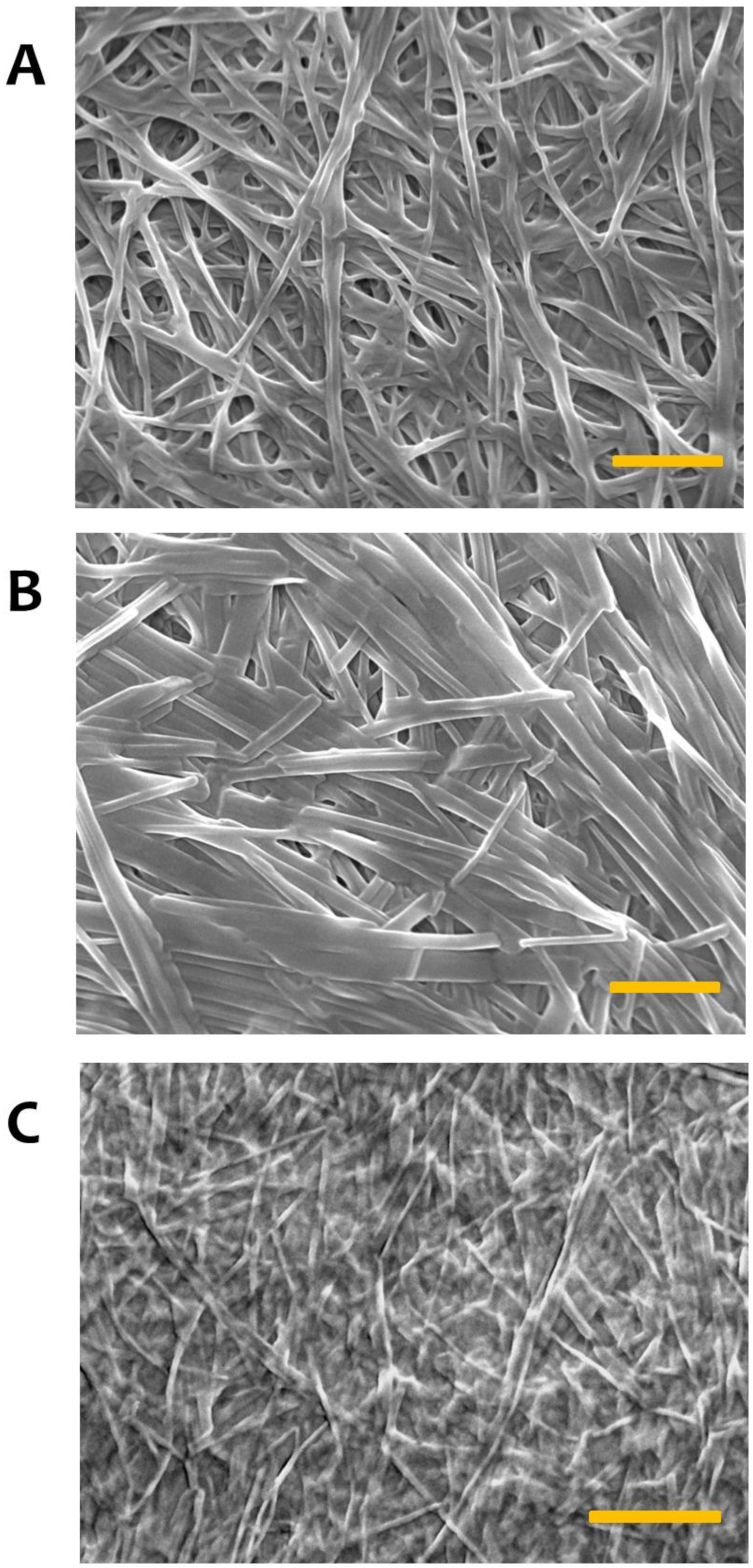

Figure S5. SEM micrographs of xerogels and made from gels at 12 mM concentration of **1**·2Br in water:ethanol ratio 5:5 (A), 7:3 (B) and 9:1 (C) at 292 K. Scale bar in all images represents 1 μm.

Table S4. Unit-cell parameters obtained from auto-indexing of diffraction pattern of xerogel 12 mM in water:ethanol ratio 7:3. The solution with the highest figure of merit (M10 = 23) displays a cell volume (1976.06 Å^3) that is comparable to theoretical one (2016 Å^3) of two molecules of 1·2Br. Moreover, one of the unit cell dimensions (a) indicates a value of 38.527 Å, which is in line with the thickness of a single bilayer obtained from the AFM analysis.

Figure of Merit	Cell Volume (Å^3)	a (Å)	b (Å)	c (Å)	α (°)	β (°)	γ (°)
23	1976.06	38.527	4.014	13.551	90	92.87	90
16.5	1975.91	38.288	4.015	13.495	90	92.85	90
15	1921.09	38.258	3.902	13.512	90	92.87	90
15	1921.08	39.527	3.902	13.512	90	92.87	90
14.5	1920.23	38.259	3.900	13.515	90	92.86	90
13.7	1919.40	38.299	3.899	13.494	90	92.84	90
13.7	1919.39	39.552	3.899	13.494	90	92.84	90

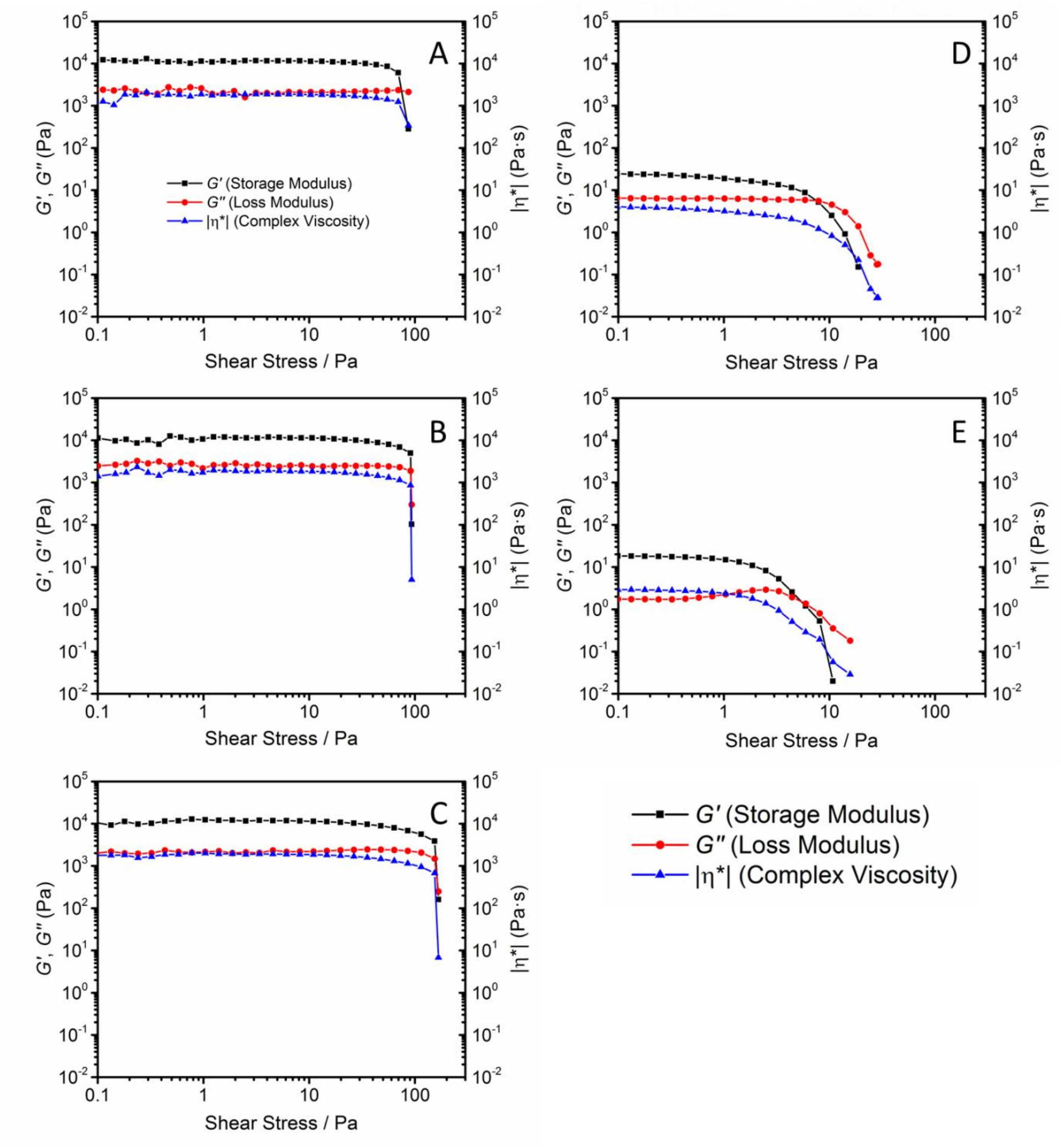

Figure S6. Shear stress profiles obtained from gels of **1**·2Br at 12 mM in water-ethanol ratio 5:5 (A), 6:4 (B) and 7:3 (C) are quite similar to one another, showing high moduli values at zero-shear stress and wide linear viscoelastic regimes, indicating that these gels have similar strength and viscosity. The main difference between these samples is the critical stress level they reach before breaking. The critical stress values (crossover points when G′ = G′′, Table S5) indicate that the gel obtained in water:ethanol ratio 7:3 (C) is significantly stronger than those at lower water content, feature that is in agreement with our qualitative observations on the macroscopic appearance of the gels and their different stability against solvent exclusion. Significant differences in the rheological behaviour were found for samples made with the water:ethanol ratios 8:2 (D) and 9:1 (E). The prevalence of the

storage modulus over the loss modulus still confirmed the solid-like response of these gels. However, G′, G′′ and complex viscosity are very significantly lower, indicating an abrupt decrease of the network strength. Moreover, these samples showed much shorter viscoelastic linear regimes and a different response to the applied tension. The critical stress levels arise at crossover values much lower than those observed for gels at lower water content, indicating a weaker but less fragile fibrillar network. Instead of a sudden rupture, an elastic behaviour exists when the crossover value is reached, indicating that gels behaved predominantly as a viscous liquid.

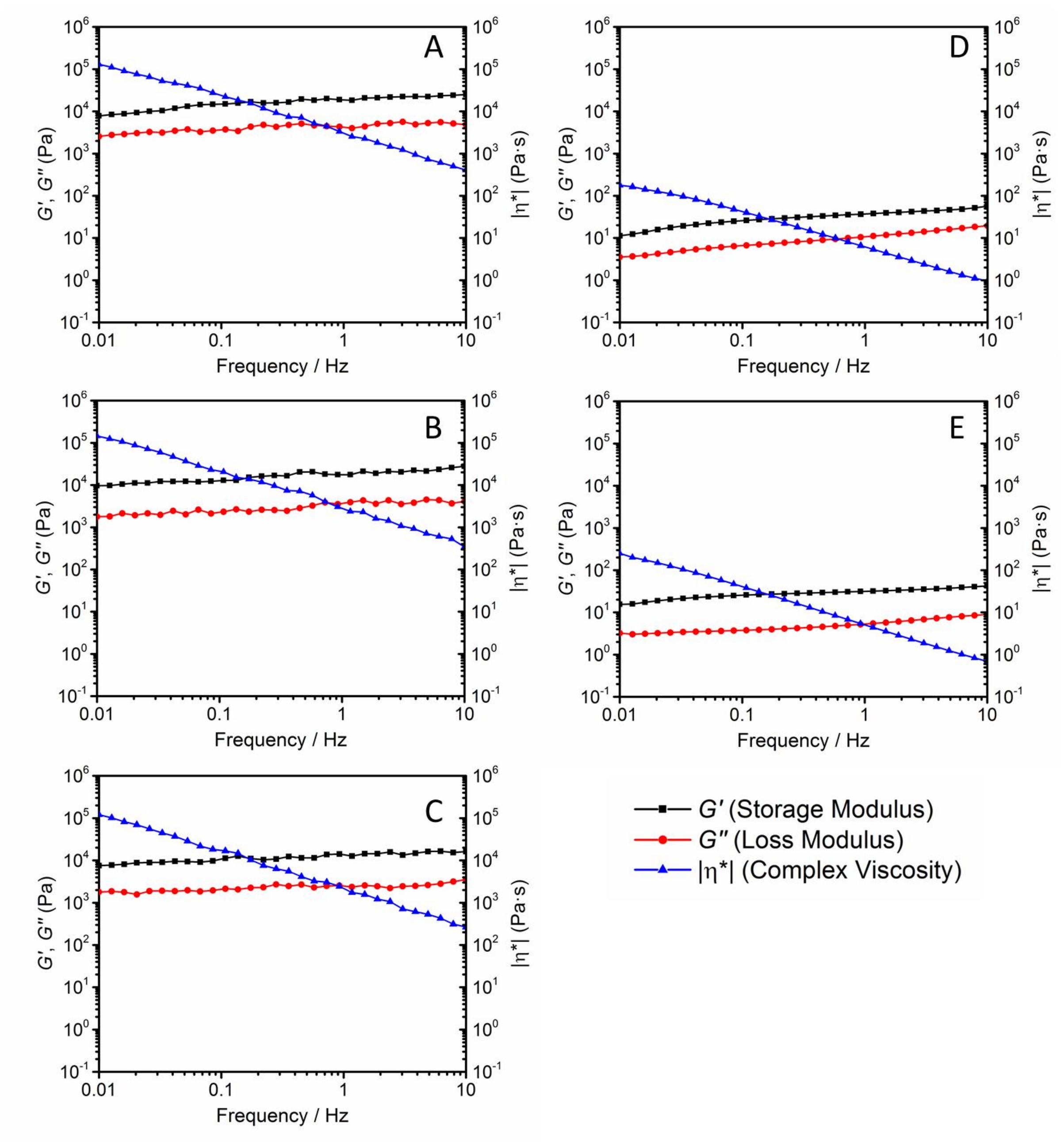

Figure S7. Frequency sweep profiles obtained from gels of **1**·2Br at 12 mM in water-ethanol ratio 5:5 (A), 6:4 (B), 7:3 (C), 8:2 (D) and 9:1 (E).

Table S5. Storage modulus (G'), loss modulus (G''), complex viscosity $|\eta^*|$ and critical stress of gels obtained from stress sweep experiments as function of water:ethanol ratio at 292 K.

Water:Ethanol ratio	G'(Pa) [a]	G''(Pa) [a]	$\|\eta^*\|$ (Pa × s) [a]	Critical Stress (Pa) [b]
5:5	11340	2120	1850	74.6
6:4	11670	2250	1880	92.1
7:3	12000	2380	1900	162.5
8:2	23.8	6.4	3.8	7.6
9:1	17.9	2.8	1.7	5.4

[a] Values at the linear viscoelastic regime.

[b] The critical stress value is defined as the cross-over point between storage and loss moduli.

Experimental Section

Materials

All reagents and solvents used in this work were of analytical grade. MilliQ water was used for the sample preparation. Compound 1,3-bis[(3-octadecyl-1-imidazolio)methyl]benzene dibromide was synthetized as previously reported in literature.[1]

Methods

Sample preparation. All mixtures were prepared by addition of water to an ethanolic solution of gelator, varying concentration of the latter from 1 to 12 mM, and water-ethanol proportion between 5:5 and 9:1. 1 mL samples were prepared in 3 mL closed vials, sealed after gentle mixing (with a micropipette) and letting them stand undisturbed at 292 K to allow the self-assembly. The vial-inversion test was used to assess gel formation. A standard preparation protocol was adopted to ensure the maximum reproducibility over all the samples: Stock solutions of gelator in ethanol were made having concentration ten times higher than those analysed. For example, the sample 12 mM in water-ethanol 9:1 was prepared by addition of 0.9 mL of water to 0.1 mL of gelator stock solution (120 mM). For the other solvent proportions, 0.1 mL of stock solution was prediluted with an appropriate amount of ethanol (0.1-0.4 mL) then water was added (0.8-0.5 mL) to a final volume of 1 mL. A sample

with concentration 12 mM and water ethanol ratio 7:3 was obtained combining 0.1 mL of stock solution 120 mM with 0.2 mL of ethanol, then 0.7 mL of water were added.

Kinetic measurements

Extinction measurements were carried out with an Agilent Cary 5000 UV-Vis-NIR spectrophotometer equipped with the Peltier 1×1 cell holder system. 10 mm quartz cuvettes were used for all the measurements. The kinetics of fibre formation was monitored following the increase of optical density at 700 nm, where no contribution to the signal comes from absorbed light is possible (the molecule absorbs in the UV region) but only by scattering from the sample, as seen in the following dataset for the 9:1 water:ethanol sample at 306 K. At lower wavelengths the scattering signal saturates the absorption spectrometer.

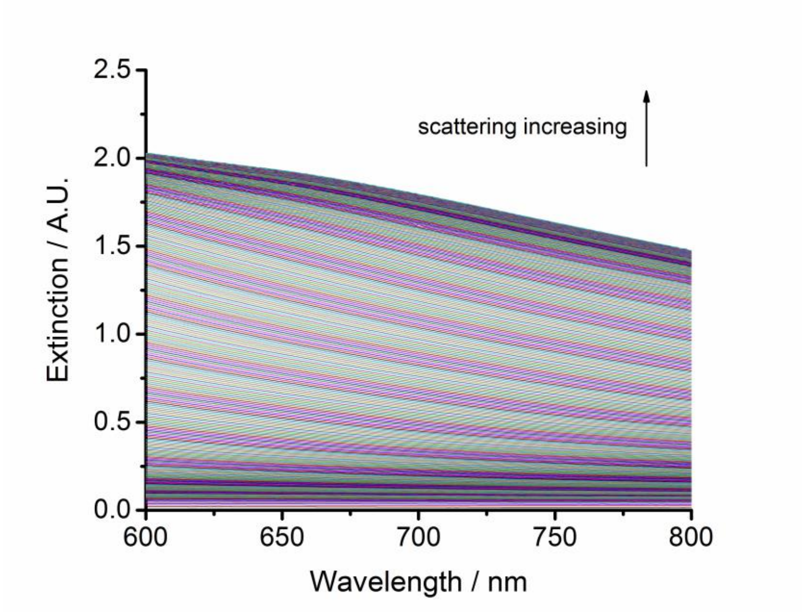

Ethanolic solutions of **1**·2Br and MilliQ water were thermally equilibrated at the desired temperature for 15 minutes before mixing. 2 mL samples were prepared using different water-ethanol proportions and a final **1**·2Br concentration of 8 mM. The mixing was done very quickly with a micropipette and then the cuvette was returned to the sample holder (generally within 2-3 s) for data acquisition. The fitting of the data to the models given in the main text was carried out using the software *Origin Pro* performing a non-linear curve fit using Levenberg Marquardt method as iteration algorithm.

Characterization

Scanning Electron Microscopy (SEM) was performed on a JEOL 7100F FEG-SEM instrument. Samples were prepared as 1mL volume in 3 mL sealed vials. Small aliquots of gel were deposited on aluminium

stubs, dried at room temperature under vacuum (fast drying achieved within 5-10 seconds after casting to minimise possible drying effects) and coated with a 5-nm thick layer of Iridium. Image acquisition was carried out using a working distance between 5-10 mm and 5 kV accelerating voltage to minimize sample charging. The rheological analysis was performed with an Anton Paar MCR 302 rheometer equipped with temperature controller and parallel plate geometry setup (PP50 stainless steel, 50 mm diameter, 1 mm gap between plates). All the measurements were carried out at 298 K. Samples were made as 6 mL volume in 7 cm diameter petri dishes and sealed to prevent solvent evaporation. Gels were analysed 2 days after preparation. Samples were carefully transferred on the rheometer plate without breakage and the extra material was removed to suit the working geometry. Flow behaviour was evaluated performing oscillation amplitude tests at deformation frequency ν = 1 Hz and shear stress τ between 0.1 and 200 Pa to determine the gels strength and the linear viscoelastic region. Atomic Force Microscopy (AFM) topographies were acquired using a Dimension FastScan Bio™ operating in peak force tapping mode, equipped with an antimony doped silicon cantilever (Bruker, type RTESPA-150 force constant 5 N/m and tip radius 8-12 nm) of resonant frequency 150–210 kHz. Samples were prepared as 1mL volume in 3 mL sealed vials. All the images were recorded under atmospheric conditions on small aliquots of sample cast on mica substrate and dried at room temperature under vacuum (fast drying achieved within 5-10 seconds after casting to minimise possible drying effects). Powder X-Ray diffraction (XRD) patterns were obtained by a PANalytical MPD X-Ray Powder Diffractometer in Bragg–Brentano geometry, using Cu-Kα radiation ($K\alpha_1$ = 1.540560 Å and $K\alpha_2$ = 1.544390 Å) with a voltage and current of 40 kV and 40 mA, respectively. 3 mL of gel was prepared in a 10 mL sealed vial for each sample analysed, which were dried under vacuum to obtain xerogels after 3 days from preparation. The powder was collected and placed on brass sample holder for data acquisition in 2-Theta scale between 2-30°, with step size of 0.013°. Diffractograms analysis for phase identification was carried out with the software CRYSFIRE suite, using Dicvol91[2] as automatic powder indexing program to obtain the unit cell. Software Xfit-Koalariet[3] was adopted for peak fitting using Pseudo-Voight functions.

References

1 L. Casal-Dujat, M. Rodrigues, A. Yagüe, A. C. Calpena, D. B. Amabilino, J. González-Linares, M. Borràs and L. Pérez-García, *Langmuir*, 2012, **28**, 2368-2381.

2 A. Boultif and D. Louer, *J. Appl. Crystallogr.*, 1991, **24**, 987-993.

3 R. W. Cheary and A. A. Coelho, *Programs XFIT and FOURYA, deposited in CCP14 Powder Diffraction Library, Engineering and Physical Sciences Research Council, Daresbury Laboratory* (1996) (http://www.ccp14.ac.uk/tutorial/xfit-95/xfit.htm).
